# Association between bisphenol A diglycidyl ether-specific IgG in serum and food sensitization in young children

**DOI:** 10.1186/s40001-018-0358-1

**Published:** 2018-12-26

**Authors:** Mayumi Tsuji, Chihaya Koriyama, Yasuhiro Ishihara, Christoph F. A. Vogel, Toshihiro Kawamoto

**Affiliations:** 1 Department of Environmental Health, University of Occupational and Environmental Health, 1-1 Iseigaoka, Yahatanishi-ku, Kitakyusyu, Fukuoka Prefecture 807-8555 Japan; 2 Department of Epidemiology and Preventive Medicine, Kagoshima University Graduate School of Medical and Dental Sciences, 8-35-1 Sakuragaoka, Kagoshima, 890-8520 Japan; 3 Laboratory of Molecular Brain Science, Graduate School of Integrated Arts and Sciences, Hiroshima University, 1-7-1 Kagamiyama, Higashi-Hiroshima, 739-8521 Japan; 4 Department of Environmental Toxicology, University of California, Davis, Davis, CA USA

**Keywords:** Bisphenol A diglycidyl ether, Children, Food sensitization, IgE, Inflammatory cytokines

## Abstract

**Background:**

Recent studies have reported that endocrine-disrupting compound (EDC) exposure is related to food sensitization. Bisphenol A diglycidyl ether (BADGE) is one of the most widespread EDCs and its biological effects are considered to be greater on children than on adults. This study investigated the relationship between serum BADGE-specific immunoglobulin G (IgG) concentrations and food sensitization in young children by measuring food-specific IgE levels.

**Methods:**

In total, 98 young children (59 boys and 39 girls; median age: 7 months; 25th and 75th percentile ages: 6 and 8 months, respectively) were enrolled. Blood samples were collected twice from all children (median sampling interval: 6 months; 25th and 75th percentile: 5 and 7 months). Food sensitization was evaluated based on food-specific IgE titers (egg white, milk, and wheat), which were determined using the capsulated hydrophilic carrier polymer-radioallergosorbent test. Furthermore, a dot-blotting assay for BADGE-specific IgG and quantitative reverse-transcriptase PCR for *IL*-*6*, *IL*-*8*, *IL*-*10*, and *COX*-*2* mRNA expression were conducted.

**Results:**

BADGE-specific IgG was detected in 20% of study subjects. A significant association was observed between the presence of BADGE-specific IgG and elevated wheat-specific IgE levels (OR = 3.56; 95% CI 1.13–11.2; *P* = 0.031). This relationship was particularly strong in girls (OR = 9.46; 95% CI 1.01–89.0; *P* = 0.049). A slight but non-significant association was noted between the presence of BADGE-specific IgG and elevated milk-specific IgE levels (OR = 2.77; 95% CI 0.93–8.22; *P* = 0.067). The expression of *IL*-*6* mRNA among children with BADGE-specific IgG tended to increase, along with wheat-specific IgE levels.

**Conclusion:**

BADGE exposure might enhance food sensitization in early childhood. Therefore, this should be strictly regulated, especially in younger children.

**Electronic supplementary material:**

The online version of this article (10.1186/s40001-018-0358-1) contains supplementary material, which is available to authorized users.

## Introduction

Bisphenol A (BPA) is produced in large quantities for use primarily in the synthesis of polycarbonate plastics and epoxy resins [1]. Its content in products has been regulated in many countries because of its estrogenic activity [1]. Bisphenol A diglycidyl ether (BADGE) is a reactive prepolymer of BPA and one of the most widely used epoxy resins in the world [2]. In contrast to BPA, the safety of BADGE has not been well evaluated and its toxicity is not well understood. Importantly, however, it has also been reported to have estrogenic activity, albeit weaker than that of BPA [3]. In our previous study, BADGE-specific IgG was identified in sera obtained from workers with contact dermatitis who had used this material in their working space [4]. This result suggested that serum BADGE-specific IgG could reflect exposure to BADGE and that exposure is related to allergic diseases.

Recent studies have reported that exposure to endocrine-disrupting compounds (EDCs) including BPA and BADGE might increase the risk of allergic diseases such as respiratory allergies, contact dermatitis, food sensitization, and food tolerance [4–7]. BPA was suggested to induce a T helper 2 (Th2)-dominated immune response through its estrogenic activity [8]. Furthermore, it was shown to have adjuvant effects on increases in ovalbumin (OVA)-specific IgE levels by promoting Th-2-immune responses, macrophage activation, and the production of inflammatory cytokines [9], suggesting that exposure to EDCs can induce the onset and/or progression of food sensitization.

BADGE is used in infant clothing, textiles, and flooring materials [10, 11]. Because infants exhibit immature behaviors such as crawling on the floor and putting random items into their mouths [1], the risk of BADGE exposure in childhood might be greater than that in adulthood. In fact, the estimated daily intake of BADGE from indoor dust is increased in children compared to that in adults, especially in Japan [12]. However, little is known about the relationship between BADGE exposure and childhood allergic diseases. For young children, transient and persistent IgE sensitization to food allergens is closely related to late childhood allergy [13]. Therefore, it is important to mitigate food sensitization in early childhood to prevent subsequent childhood allergies. In young Japanese children, egg white, milk, and wheat are the most common allergens [14]. In this study, we investigated the relationship between serum BADGE-specific IgG levels and egg white, milk, and wheat sensitization in young children.

## Materials and methods

### Subjects and questionnaires

Children aged 6 months–1 year and their parents were recruited at a single clinic between January 2009 and February 2010. In total, 115 mothers of 140 children provided informed consent to participate in the study (consent rate: 82%). Nine mothers could not recall their children’s birth weight and blood samples could not be obtained from one child because of exceptionally thin blood vessels. Seven children were older than 1 year of age. Thus, the final number of subjects for analysis was 98 children [59 boys (median age: 7 months) and 39 girls (median age: 7 months]. The questionnaire administered at the maternal interview included questions concerning birth weight, feeding, number of siblings, allergy history, parental smoking habits, and parental allergy history. The survey was conducted at the time of first blood sample collection.

### Blood collection

Blood samples were collected from children without fever, allergic symptoms, infectious diseases, and skin eczema, at least 1 month prior to sampling. Blood sampling for children with these symptoms was postponed until 1 month following the disappearance of symptoms. Blood samples were collected twice from all children, and the median blood sampling interval was 6 months (25th and 75th percentile: 5 and 7 months). The first blood sampling was conducted approximately 6 months after birth because children in Japan typically begin eating solids at that age [14]. In addition, antibody-specific IgE tests for children under 6 months of age often present inaccurate results [14]. According to the food allergy guidelines for children, the standard interval for IgE measurements is every 6 months for children under 3 years of age and every 6–12 months for oral food-challenge tests [15]. Therefore, we conducted the second sampling between 6 and 12 months after the first blood sampling. Approximately 3–5 mL of blood was collected at each sampling.

### Allergen-specific IgE assays

Egg white-, milk-, and wheat-specific IgE levels were determined using the capsulated hydrophilic carrier polymer-radioallergosorbent test (CAP-RAST; FALCO Biosystems Ltd., Kyoto, Japan). Both the first and second blood samples were subjected to CAP-RAST to examine changes in response levels to these antigens. For this, 0.3 mL of serum was used to evaluate each allergen. The detection of IgE levels greater than 0.35 UA/mL was considered as a sign of sensitization [16]. Therefore, subjects presenting with values ≥ 0.35 UA/mL were placed in the food-specific IgE-positive group and those with values of < 0.35 UA/mL were assigned to the food-specific IgE-negative group.

### Detection of BADGE-specific IgG by dot blotting

BADGE-specific IgG concentrations were measured using only the first-collected blood sample in accordance with our previously reported protocol [4, 17, 18]. Briefly, BADGE (Sigma-Aldrich., St. Louis, MO, USA) and human serum albumin (HSA; Sigma-Aldrich) were mixed at a 1:100 ratio at pH 10.8 to form an HSA-BADGE adduct, which was used as an antigen. The antigens and human IgG (positive control; Zymed Laboratories, Inc., San Francisco, CA, USA) were electroblotted onto nitrocellulose membranes (Amersham, GE Healthcare, Japan) and blocked. The membranes were probed with serum (1:200 dilution) and subsequently treated with horseradish peroxidase (HRP)-conjugated goat anti-human IgG (Millipore, Japan; 1:4000 dilution), which was followed by visualization.

### Quantitative real-time PCR (qRT-PCR)

IL-6, IL-8, and IL-10 are involved in IgE production and immunological tolerance [19–23]. COX-2 has been reported to suppress IgE production during Th2 responses in allergic children [24]. Using the first-collected blood samples, mRNA levels of genes encoding these cytokines were measured by qRT-PCR as reported in our previous studies [25, 26]. Whole blood was collected in a heparin-coated tube under a vacuum and maintained at 4 °C. Total RNA was isolated using a QIAamp RNA Blood Mini kit (Qiagen, Hilden, Germany). RNA was reverse-transcribed into cDNA using a Quanti Tect Reverse Transcription kit (Qiagen) and quantitative detection was performed using a Step One Plus Real-Time PCR System (Applied Biosystems, Inc., Foster City, CA, USA) with Fast SYBR Green Master Mix (Applied Biosystems Inc.). The sequences of primers used in this study are shown in Additional file 1: Table S1.

### Statistical analyses

The study subjects were divided into two groups based on food-specific IgE levels, specifically negative (< 0.35 UA/mL) and positive (≥ 0.35 UA/mL), to examine the association between this classification and the presence of BADGE-specific IgG. Individual changes in food sensitization were also examined by determining the difference of IgE titers between the first and second collections [2nd sample IgE value (UA/mL) − 1st sample IgE value (UA/mL)]. Based on the calculation of this difference, the subjects were then further divided into two groups as decrease/no change (≤ 0 UA/mL) and increase (> 0 UA/mL).

Two-group comparisons were performed using Mann–Whitney U tests and multivariable logistic regression analysis. Adjusted *P* values were obtained by multivariable logistic regression models using age at first CAP-RAST, feeding, and allergic diseases (wheezing, allergic rhinitis, and atopic dermatitis) as covariates. Because a significant sex-specific difference in the effects of EDCs on the immune response was previously reported [6, 27], boys and girls were also separately analyzed.

All analyses were performed using STATA version 14 (Stata Corporation, Inc., College Station, TX, USA), and statistical significance was assumed when *P* < 0.05 (two sided).

## Results

Table 1 shows the characteristics of the study subjects. The median age (25th and 75th percentile) at first blood collection was 7 (6, 8) months for both boys and girls. There was no significant difference in the distribution of the evaluated variables between boys and girls. When BADGE-specific IgG (> 0 µg/mL) was detected, the subject was assigned to the “detected” group. The BADGE-IgG detection rate in subjects was 20%. The median (range) BADGE-specific IgG concentrations in boys and girls of the detected groups were 0.57 (0.03–12.4) and 0.35 (0.04–3.85) µg/mL, respectively. There was no significant association between the detection of BADGE-specific IgG in children and the allergic history of parents (none vs either or both parents) by Fisher’s exact test (*P* = 1.000).

**Table 1:** Characteristics of study subjects

	Number (%)			*P* value^‡^
	All	Boys	Girls	
Number	98 (100)	59 (100)	39 (100)	
Birth weight (g)				
< 2500	9 (9)	4 (7)	5 (13)	
≥ 2500	89 (91)	55 (93)	34 (87)	0.477
Feeding				
Breast milk	52 (53)	29 (49)	23 (59)	
Breast milk/formula	46 (47)	30 (51)	16 (41)	0.410
Number of siblings				
0	56 (57)	33 (56)	23 (59)	
1	32 (33)	20 (34)	12 (31)	0.823
≥ 2	10 (10)	6 (10)	4 (10)	1.000
Wheezing				
No	78 (79)	48 (81)	30 (77)	
Yes	20 (20)	11 (19)	9 (23)	0.617
Atopic dermatitis				
No	89 (91)	52 (88)	37 (95)	
Yes	9 (9)	7 (12)	2 (5)	0.310
Allergic rhinitis				
No	89 (91)	52 (88)	37 (95)	
Yes	9 (9)	7 (12)	2 (5)	0.310
Smoking habits of parents				
No	42 (43)	27 (46)	15 (38)	
Yes	56 (57)	32 (54)	24 (62)	0.535
Allergic history of parents				
None	11 (11)	5 (8)	6 (15)	
Either	57 (58)	35 (59)	22 (56)	0.341
Both	30 (31)	19 (32)	11 (28)	0.476
BADGE-specific IgG				
Not detected	78 (80)	47 (80)	31 (79)	
Detected^†^	20 (20)	12 (20)	8 (21)	0.269

^†^Detected: > 0 µg/mL

^‡^*P* values were obtained by performing a Fisher’s exact test

The number of subjects positive for food-specific IgE and the change in serum IgE levels between the first and second tests are shown in Table 2. For 93% (*N* = 91) of the subjects, the second blood sampling was performed between 6 and 12 months after the first blood collection, and the remaining 7% (*N* = 7) of samples were collected between 1 and 2 years after the first sampling. The frequencies of positive subjects based on the first CAP-RAST were 74%, 32%, and 19% for egg white-, milk-, and wheat-specific IgE, respectively.

**Table 2:** Distribution of food-specific IgE levels and change in IgE levels between first and second blood collections

	Number (%)			*P* value^§^
	All	Boys	Girls	
First CAP-RAST^†^				
Egg-specific IgE				
Positive	73 (74)	44 (75)	29 (74)	1.000
Milk-specific IgE				
Positive	31 (32)	17 (29)	14 (36)	0.510
Wheat-specific IgE				
Positive	19 (19)	15 (25)	4 (10)	0.073
Second CAP-RAST^†^				
Egg-specific IgE				
Positive	82 (84)	49 (83)	33 (85)	1.000
Milk-specific IgE				
Positive	37 (38)	22 (37)	15 (38)	1.000
Wheat-specific IgE				
Positive	31 (32)	21 (36)	10 (26)	0.377
Change in CAP-RAST values^‡^				
Egg-specific IgE				
Decrease or no change	62 (63)	40 (68)	22 (56)	
Increase	36 (37)	19 (32)	17 (44)	0.289
Milk-specific IgE				
Decrease or no change	75 (77)	47 (80)	28 (72)	
Increase	23 (23)	12 (20)	11 (28)	0.466
Wheat-specific IgE				
Decrease or no change	78 (80)	45 (76)	33 (85)	
Increase	20 (20)	14 (24)	6 (15)	0.443

*CAP-RAST* carrier polymer-radioallergosorbent test

^†^Negative: < 0.35; positive: ≥ 0.35 UA/mL

^‡^Decrease/no change: [2nd IgE value (UA/mL) − 1st IgE value (UA/mL)] ≤ 0, Increase: [2nd IgE value (UA/mL) − 1st IgE value (UA/mL)] > 0

^§^*P* values were obtained by performing a Fisher’s exact test

The relationship between the presence of BADGE-specific IgG and food-specific IgE titers at the first CAP-RAST was also examined, but no food-specific IgE was related to the presence of BADGE-specific IgG (Table 3).

**Table 3:** Relationship between BADGE-specific IgG detection and food-specific IgE levels at first carrier polymer-radioallergosorbent test (CAP-RAST)

BADGE-specific IgG	Egg white-specific IgE			*P* value^‡^	Milk-specific IgE			*P* value^‡^	Wheat-specific IgE			*P* value^‡^
	Negative^†^	Positive^†^	OR (95% CI)		Negative^†^	Positive^†^	OR (95% CI)		Negative^†^	Positive^†^	OR (95% CI)	
All												
Not detected	21	57	1.00 (reference)		53	25	1.00 (reference)		62	16	1.00 (reference)	
Detected	4	16	1.42 (0.41–4.89)	0.580	14	6	0.85 (0.28–2.53)	0.764	17	3	0.60 (0.15–2.40)	0.470
Boys												
Not detected	12	35	1.00 (reference)		33	14	1.00 (reference)		35	12	1.00 (reference)	
Detected	3	9	1.02 (0.21–4.98)	0.977	9	3	0.59 (0.12–2.89)	0.518	9	3	0.82 (0.18–3.76)	0.793
Girls												
Not detected	9	22	1.00 (reference)		20	11	1.00 (reference)		27	4	1.00 (reference)	
Detected	1	7	2.69 (0.27–26.8)	0.400	5	3	1.22 (0.23–6.49)	0.819	8	0	–	

^†^Negative: < 0.35, positive: ≥ 0.35 (UA/mL)

^‡^*P* values were obtained using multivariable logistic regression analysis adjusted for age at first CAP-RAST, feeding, and allergic diseases (wheezing, allergic rhinitis, atopic dermatitis)

Table 4 shows the relationship between BADGE-specific IgG detection and the change in IgE titers between the first and second tests. All subjects with BADGE-specific IgG showed a significant increase in wheat-specific IgE (OR = 3.56; 95% CI, 1.13–11.2; *P* = 0.031). This relationship was also significant when the subjects were limited to those from whom the second blood sample was collected within one year of first sampling (*N* = 91, OR = 3.97; 95% CI, 1.23–12.9; *P* = 0.021). Furthermore, this association was more significant in girls (OR = 9.46; 95% CI, 1.01–89.0; *P* = 0.049); however, the interaction with sex was not statistically significant (*P *= 0.535). All subjects with BADGE-specific IgG tended to show an increase in milk-specific IgE levels (OR = 2.77; 95% CI, 0.93–8.22; *P* = 0.067), although this association was not statistically significant.

**Table 4:** Relationship between BADGE-specific IgG detection and change in food-specific IgE values

BADGE-specific IgG	Egg white-specific IgE			*P* value^‡^	Milk-specific IgE			*P* value^‡^	Wheat-specific IgE			*P* value^‡^
	Decrease/no change^†^	Increase^†^	OR (95% CI)		Decrease/no change^†^	Increase^†^	OR (95% CI)		Decrease/no change^†^	Increase^†^	OR (95% CI)	
All												
Not detected	50	28	1.00 (reference)		63	15	1.00 (reference)		66	12	1.00 (reference)	
Detected	12	8	1.22 (0.41–3.59)	0.722	12	8	2.77 (0.93–8.22)	0.067	12	8	3.56 (1.13–11.2)	0.031
Boys												
Not detected	33	14	1.00 (reference)		40	7	1.00 (reference)		38	9	1.00 (reference)	
Detected	7	5	2.16 (0.50–9.44)	0.304	7	5	4.93 (0.89–27.2)	0.067	7	5	2.77 (0.65–11.8)	0.167
Girls												
Not detected	17	14	1.00 (reference)		23	8	1.00 (reference)		28	3	1.00 (reference)	
Detected	5	3	0.44 (0.07–2.86)	0.391	5	3	2.14 (0.35–13.2)	0.412	5	3	9.46 (1.01–89.0)	0.049

^†^Decrease/no change: [IgE value at 2nd (UA/mL) − IgE value at 1st (UA/mL)] ≤0; increase: [IgE value at 2nd (UA/mL) − IgE value at 1st (UA/mL)] > 0

^‡^*P* values were obtained using multivariable logistic regression analysis adjusted for age at first carrier polymer-radioallergosorbent test (CAP-RAST), feeding, and allergic diseases (wheezing, allergic rhinitis, atopic dermatitis)

**Fig. 1:**
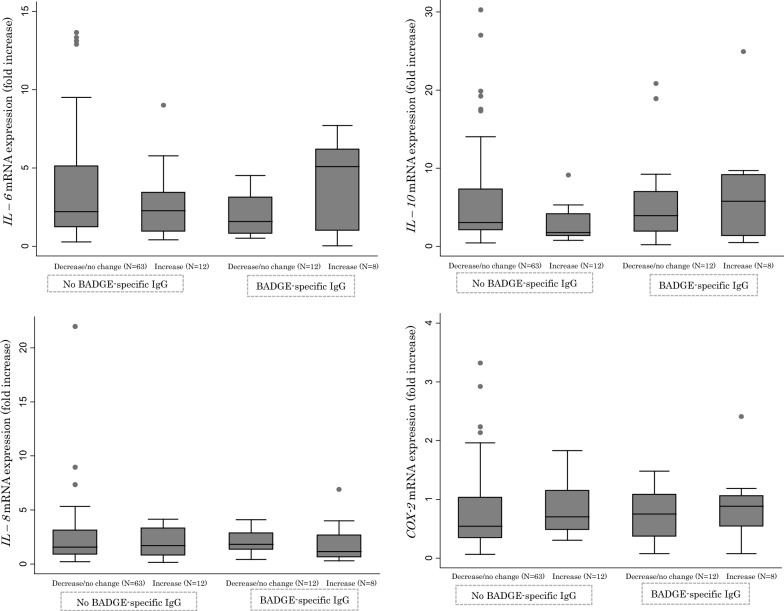
Relationship between mRNA expression and changes in wheat-specific IgE values between the first and second blood samples in groups with or without detected BADGE-specific IgG. Shown is a box plot bordered at the 25th and 75th percentiles of the variable on the *y*-axis with a median line at the 50th percentile for the expression of each mRNA

To examine the immunological responses related to IgE production, *IL*-*6*, *IL*-*8*, *IL*-*10*, and *COX2* expression was examined. Figure 1 shows the comparison of mRNA expression levels between the group with increased wheat-specific IgE and others according to the presence of BADGE-specific IgG. Three subjects were not examined due to insufficient blood samples. No significant relationships were identified between the expression of cytokines and COX-2 based on a Mann–Whitney *U* test, but the subjects with both BADGE-specific IgG and increased wheat-specific IgE levels presented an approximate two- to threefold increase in median *IL*-*6* mRNA expression, compared to the other groups.

## Discussion

In the present study, the BADGE-IgG detection rate was 20%. A significant increase in risk for elevated wheat-specific IgE levels was observed among children with BADGE-specific IgG, and this association was more significant in girls than in boys, although the interaction with sex was not statistically significant. There was no significant difference between BADGE-specific IgG status in children and the allergic history of parents. There are no previous studies describing the relationship between BADGE-specific IgG and the allergic history of parents. Therefore, this study is the first to indicate that the allergic history of parents does not influence BADGE-specific IgG in children. In future studies, the allergic history of parents should be included when measuring childhood BADGE-specific IgG.

The subjects with both BADGE-specific IgG and increased wheat-specific IgE levels presented with increased *IL*-*6* mRNA expression compared to the other groups. Serum BADGE-specific IgGs probably reflect BADGE exposure even though the association with dose–response is unclear [4, 28]. In our previous study [4], 90% (9 of 10) of workers who had used epoxy resin and developed contact dermatitis had BADGE-specific IgG. In contrast, 13% (8 of 104) of adults without occupational BADGE exposure harbored BADGE-specific IgG, which was also detected by the same dot blot analysis (unpublished data). Thus, the results of the present study suggest that food sensitization among young children might be enhanced by BADGE exposure in their daily lives.

As stated, up-regulation of IL-6, a Th2-related cytokine, was detected in the group presenting both elevated wheat-specific IgE levels and detectable BADGE-specific IgG. Considering that BADGE has estrogenic activity, although not to the same extent as BPA [3], and that BPA has been suggested to induce a Th2 immune response through its estrogenic activity [8], BADGE exposure might elicit a Th2 immune response via IL-6 upregulation through its estrogenic activity. In addition, exposure to BADGE induces Toll-like receptor (TLR) 4 expression in rat macrophages [29], and this receptor might drive a Th2 immune response [30]. Therefore, the expression of TLR4 in response to BADGE exposure might be one cause of the observed increase in food-specific IgE levels. Further experiments are needed to verify this.

The association between the presence of BADGE-specific IgG and the increase in wheat specific-IgE levels was stronger among girls than among boys, although this interaction was not statistically significant. The effect of BPA exposure was previously shown to vary based on sex, wherein female mice showed a consistently enhanced Th2 response upon BPA exposure, compared to that in male mice [31, 32]. Similarly, in the present study, girls in the BADGE-IgG-detected group showed slightly higher mRNA expression levels of *IL*-*6*, which encodes a Th2-related cytokine (median: 2.54; 25th and 75th percentile: 0.63 and 4.64, respectively), compared to the boys (median: 1.58; 25th and 75th percentile: 1.00 and 5.41, respectively). However, BADGE-specific IgG levels in girls were lower than those in boys; the median (range) BADGE-specific IgG levels in boys and girls of the BADGE-detected groups were 0.57 (0.03–12.4) and 0.35 (0.04–3.85) µg/mL, respectively. These results suggest that the reaction to BADGE exposure is stronger in girls than in boys.

The present study has some limitations that merit discussion and further research. First, in this study, a significantly increased risk of elevation in wheat-specific IgE levels was observed among children with detected BADGE-specific IgG. For the other two food-specific IgEs, namely egg white and milk, a similar tendency was also observed, although these associations were not statistically significant (Table 4). There is currently no explanation for why a significant association was observed only for wheat-specific IgE. Individuals in our cohort were not subjected to food limitation, and we did not obtain information regarding their nutrition intake. Further studies employing a food-frequency questionnaire are required to reveal whether BADGE exposure is involved in food-specific IgE increases and subsequently enhanced sensitization to specific foods. Second, to comprehensively measure cytokines, we detected mRNA expression levels using qRT-PCR. Enzyme-link immunosorbent assay (ELISA) is another method to detect cytokines. Trends for cytokine profiles based on measuring mRNA expression by qRT-PCR are generally the same as those determined by ELISA [33–35]. However, unlike ELISA, cytokine mRNA expression levels do not always correspond to actual protein levels. Future studies will also incorporate the quantification of proteins by ELISA into these protocols and more quantitative studies will be performed.

## Conclusion

This is the first study investigating the relationship between BADGE-specific IgG and food-specific IgE sensitization in early childhood. Our study suggests that exposure to this material might affect food sensitization in early childhood. Therefore, BADGE exposure should be strictly regulated, especially in younger children.

## Additional file

**Additional file 1: Table S1.** List of real-time PCR primers used in this study.
