# Supplementary material for: Association between bisphenol A diglycidyl ether-specific IgG in serum and food sensitization in young children
Source: Eur J Med Res. 2018 Dec 26;23:61. doi: 10.1186/s40001-018-0358-1 (PMC6306001; doi:10.1186/s40001-018-0358-1)
Supplement: Supplementary file 1 — Additional file 1: Table S1. List of real-time PCR primers used in this study. [file 40001_2018_358_MOESM1_ESM.docx]

**Additional Table S1. List of real-time PCR primers used in this study**

|  | Forward (5′-3′) | Reverse (5′-3′) |
| --- | --- | --- |
| IL-6 | GAAGGCAGCAGGCAACAC | CAGGAGCCCAGCTATGAACT |
| IL-8 | CTGCGCCAACACAGAAATTA | ATTGCATCTGGCAACCCTAC |
| IL-10 | TGGGGGAGAACCTGAAGAC | CCTTGCTCTTGTTTTCACAGG |
| COX-2 | TGAAACCCACTCCAAACACA | GAGAAGGCTTCCCAGCTTTT |
| GAPDH | GAGTCAACGGATTTGGTCGT | TTGATTTTGGAGGGATCTCG |
